# Shape of the self-concept clarity change during group psychotherapy predicts the outcome: an empirical validation of the theoretical model of the self-concept change

**DOI:** 10.3389/fpsyg.2015.01598

**Published:** 2015-10-27

**Authors:** Rafał Styła

**Affiliations:** Department of Psychology, University of WarsawWarsaw, Poland

**Keywords:** psychotherapy, processes of change, process-outcome research, self-concept clarity, shape of change, dynamic systems theory, assimilation, accommodation

## Abstract

**Background:** Self-Concept Clarity (SCC) describes the extent to which the schemas of the self are internally integrated, well defined, and temporally stable. This article presents a theoretical model that describes how different shapes of SCC change (especially stable increase and “V” shape) observed in the course of psychotherapy are related to the therapy outcome. Linking the concept of Jean Piaget and the dynamic systems theory, the study postulates that a stable SCC increase is needed for the participants with a rather healthy personality structure, while SCC change characterized by a “V” shape or fluctuations is optimal for more disturbed patients.

**Method:** Correlational study in a naturalistic setting with repeated measurements (*M* = 5.8) was conducted on the sample of 85 patients diagnosed with neurosis and personality disorders receiving intensive eclectic group psychotherapy under routine inpatient conditions. Participants filled in the Self-Concept Clarity Scale (SCCS), Symptoms' Questionnaire KS-II, and Neurotic Personality Questionnaire KON-2006 at the beginning and at the end of the course of psychotherapy. The SCCS was also administered every 2 weeks during psychotherapy.

**Results:** As hypothesized, among the relatively healthiest group of patients the stable SCC increase was related to positive treatment outcome, while more disturbed patients benefited from the fluctuations and “V” shape of SCC change.

**Conclusions:** The findings support the idea that for different personality dispositions either a monotonic increase or transient destabilization of SCC is a sign of a good treatment prognosis.

## Introduction

Many theories of therapy (e.g., cognitive and person-centered) stress the importance of self-concept change in the process of effective psychotherapy (Corsini and Wedding, 2004). However, very little is known about the shapes of self-concept change that should be observed in the course of therapy. To extend the knowledge in this field, the model of self-concept change in psychotherapy is presented and validated. The model is inspired, among others, by the Piagetian concepts of assimilation and accommodation (Piaget, 1970) and dynamic systems theory applied to psychotherapy research (Hayes and Strauss, 1998). It covers the topic of different patterns of the self-concept change and their relation to psychotherapy effectiveness. Self-concept in the current study is operationalized by the notion of self-concept clarity (SCC; Campbell et al., 1996). The main objective of the study is to answer the question “What shape of SCC change for what type of patients is correlated with positive psychotherapy outcomes?” To address this question, individual time course data on SCC change during group psychotherapy of patients with neurosis, personality disorders, or both were gathered and analyzed.

### Self-concept clarity and psychological adjustment

Self-concept can be defined as a complex cognitive schema that organizes one's knowledge about oneself (e.g., Greenwald and Pratkanis, 1984). In the current scientific literature, there is an important distinction between the content and the structure of the self-concept (Campbell et al., 2003). The content includes all the information that people gather about themselves and the way they evaluate it. This allows them to answer such questions as “Who am I?” and “How do I evaluate myself?” The structure of the self-concept refers to the architecture of the self-schema. Two important features are the level of structure integration (also known as “structure unity”) and the level of structure pluralism (Campbell et al., 2003).

SCC is one of the conceptualizations that describe structural integration of the self-concept. Campbell et al. define SCC as “the extent to which the contents of an individual's self-concept (e.g., perceived personal attributes) are clearly and confidently defined, internally consistent, and temporally stable” (Campbell et al., 1996, p. 141). There is much empirical evidence that SCC is related to psychological adjustment (for review, see Campbell et al., 2003). SCC correlates positively with self-esteem, positive affect, and extraversion, and negatively with depression, anxiety, neuroticism (Campbell et al., 1996, 2003), and symptoms of neurosis (Styła, 2011). Research done by Huflejt-Łukasik (2010) indicates that people diagnosed with depression and paranoid schizophrenia had significantly lower SCC than the healthy control group. A recent study has shown also that SCC can be increased after psychotherapy (Roepke et al., 2011).

### Model of the self-concept change in psychotherapy

Many schools of psychotherapy consider self-concept change a central aspect of an effective therapeutic process (Corsini and Wedding, 2004). Cognitive therapy deals with maladaptive assumptions about oneself and the outer world. The schemas are treated as testable hypotheses that the patient can examine through exploration and behavioral experiments and decide if they are accurate or adaptive (Beck and Weishaar, 2004). Person-centered therapy sees the inconsistencies in the organization of the self as a key source of client's problems. The therapist tries to initiate constructive changes that should lead to greater self-concept integration thanks to an attitude toward the client that is characterized by genuineness, unconditional positive regard, and empathy (Raskin and Rogers, 2004).

Empirical studies on psychotherapy effectiveness report that effective psychotherapy leads to the positive change in the content of self-schemas (e.g., Sobański et al., 2014; Vreeswijk et al., 2014) and higher self-concept integration (Strauman et al., 2001; Rakowska, 2006; Elliott, 2007; Pemberton, 2009; Roepke et al., 2011; Styla, 2012). In accordance with this empirical evidence, the model of the self-concept change presented in this section states that an effective psychotherapy positively influences the content and the structure of the self-concept. “Positively” means that the content becomes more functional and the structure becomes more integrated. However, do all the patients need the same level of change in self-concept content? The literature suggests that there might be significant differences in the needs of the patients. We know, for instance, that people with personality disorders have maladaptive self-schemas that consist of inflexible, pervasive, dysfunctional beliefs about the self and the world (Young, 1994). Effective therapy among this group of patients necessitates deep self-concept restructuring by changing the maladaptive self-schemas into more functional ones (Young, 1994; Vreeswijk et al., 2014). People with an Axis I diagnosis have significantly more adaptive self-schemas than those with an Axis II diagnosis (Lee et al., 1999), and for that reason the level of needed change in the self-concept content also differs between these two groups (Young, 1994). The model of the self-concept change postulates that the patients with maladaptive self-schemas need a deep self-concept restructuring that can be presented as a result of accommodation process (Piaget, 1970), while smaller alteration in the self-concept schema are more suitable for the less disturbed patients that can be framed in the Piagetian concept of assimilation (Piaget, 1970).

#### What are the assimilation and accommodation processes?

The concepts of assimilation and accommodation were already incorporated in a model of change in psychotherapy called the “assimilation model” (Stiles et al., 1990). Stiles et al. (1990) understand assimilation and accommodation as a joint process and they do not propose different predictions connected with them. As it will appear further the differentiation between those two processes is crucial for the model presented in this paper.

Assimilation and accommodation, according to the theory of Piaget (1970), are two processes that allow the individual to adapt to the environment through the change of structures in the mind (van Geert, 1998). Piaget broadly defined assimilation as “the integration of external elements into evolving or completed structures of an organism” (Piaget, 1970, pp. 706–707). It means that the incoming internal or external stimulus is understood and shaped by the already existing cognitive structures. Accommodation is “any modification of an assimilatory scheme or structures by the elements it assimilates” (Piaget, 1970, p. 708). While assimilation is conservative and allows the sense of continuity, accommodation makes the change of a structure possible. It leads to a transformation of the existing structures based on the encountered new elements.

It is important to note that these two processes are not absolutely separated. All acts of cognitive processing should be perceived as a simultaneous equilibration of assimilation and accommodation. As Piaget put it, “There is no assimilation without accommodation. Accommodation does not exist without simultaneous assimilation either” (Piaget, 1970, p. 708). So when we say the process of assimilation has occurred, one should rather have in mind that assimilation has outweighed accommodation; the reverse may also occur. If we say accommodation, we mean it prevails over assimilation. Therefore, this is a matter of a ratio of one process to another (Block, 1982).

#### When do people assimilate and when do they accommodate?

In his work, Block was devoted to some reformulations of Piagetian theory of adaptation and coined the principle “Assimilate if you can, accommodate if you must!” (Block, 1982, p. 286). He argued that as an effect of evolution, assimilation for human beings is the first choice strategy because it preserves the existing state of equilibrium of cognitive structure and so is more adaptively economic than accommodation. Accommodation is the second line of adaptation when assimilation of new incoming elements fails. Then, it is more adaptively effective to create new structures that will allow future assimilation of that which before was inassimilable.

Cognitive schemas may be adaptive or dysfunctional (Beck and Weishaar, 2004). Dysfunctional schemas can be defined as rigid, enduring, and maladaptive beliefs about oneself, one's relationships with others, and the world (Kriston et al., 2012). In accordance with the principle of Block (1982), the prevalence of assimilative or accommodative processes during psychotherapy should depend on the level of initial functionality of self-concept contents. Some patients come to therapy with the content of self-schemas that is close to that of the healthy population, while other's content of the self-concept is heavily disturbed (Kriston et al., 2013). Thus, the first group of patients—called “functional” in the model of self-concept change—can assimilate new knowledge about themselves without changing their self-image drastically. They do not have to accommodate (and they do not need to), because the self-concept content is not that dysfunctional and probably not far away from the feedback and interpretations they hear in psychotherapy. The second group is called “dysfunctional.” Patients from this group must accommodate incoming feedback as their actual self-schema leads them to great troubles and their self-knowledge is probably in strong opposition to the information they gather about themselves during psychotherapy. Therefore, the existing cognitive structures do not allow the encountered elements to be assimilated and the adaptation is not optimal.

#### How to distinguish assimilation from accommodation?

The next question that I would like to address is “What are the markers of assimilation and accommodation?” van Geert (1998) analyzed this question using the perspective of dynamic systems theory^1^. van Geert (1998) proves through theoretical explanations and computer simulations that assimilation has a stabilizing role for the system (e.g., cognitive structures). Assimilation can be presented as a process of widening already existing stable attractor by the new, but not revolutionary, information (Nowak and Vallacher, 1998). The notion of attractor in dynamic systems theory describes a system in which the trajectories are “attracted” to one or more points in the phase space. The concept of attractor can be visualized as a valley in the landscape, where everything (like stones, water, and snow) tends to go down to the bottom. That is why—as van Geert (1998) proves—the changes acquired through assimilation have a continuous trajectory. The already existing attractor becomes bigger and bigger, which has a stabilizing role for the system. Accommodation, however, can be visualized as a situation in which the old stable attractor is challenged by emergence of a new attractor (Nowak and Vallacher, 1998). The growth of a new attractor leads to the transition from one to the other state of the system through a period of phase transition (also called bifurcation). Transition is a period of time when the two states of the system (two attractors, e.g., old and new view of oneself) are equally possible what is a source of instability.

Taking into account conclusions drawn from the work of Piaget (1970), Block (1982), and van Geert (1998), the model of the self-concept change postulates that an assimilative process of SCC should be marked by a monotonic growth without periods of amplified variability. New elements encountered during psychotherapy perceived through the existing self-schemas should extend and clarify self-concept, leading to a greater internal consistency and temporal stability. In the process of accommodation, however, I expect to see a period of self-concept destabilization in a form of amplified fluctuations of the SCC and a period of decreased SCC in a “V” shape form. New information coming to the patient during psychotherapy that is in opposition to the existing self-concept schemas creates a new, competing equilibrium for the system. During this period of transition the old and new knowledge “fight” for the attention that impedes one's clear view of self and produces internal conflicts. But with time passing, the new equilibrium wins that which leads to greater self-concept stability and internal consistency, something that should be marked by an increase of SCC. In accordance with these postulates, Cummings et al. (2012) and Hayes and Yasinski (2015) proved that temporal destabilization in the self during cognitive therapy is a positive predictor of improvement in personality disorder and depression symptoms at the end of treatment among patients with avoidant and obsessive–compulsive personality disorder.

#### Four categories of patient characteristics

**Figure 1:**
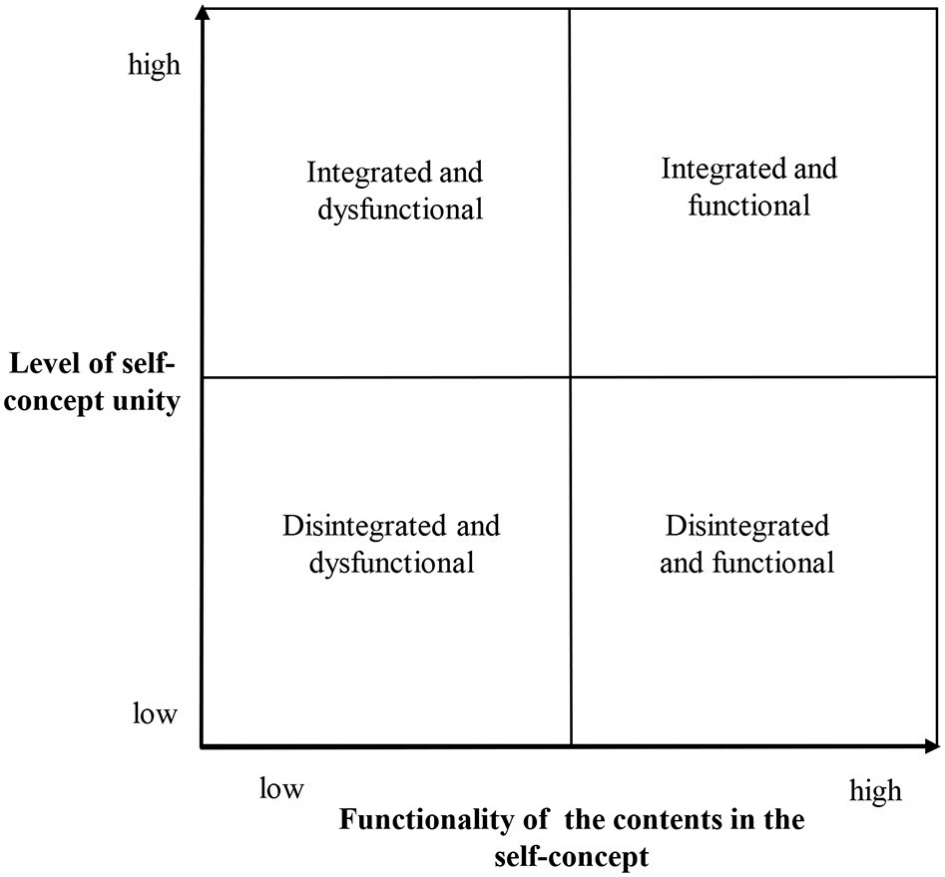
**Four categories of personality dispositions**.

There is a second important dimension of patient characteristics that is proposed in the described model—the level of initial self-concept unity. Based on the two dimensions (self-concept integration and the functionality of content of the self-concept) it is proposed to distinguish four types of personality disposition: (1) integrated and functional, (2) integrated and dysfunctional, (3) disintegrated and functional, (4) disintegrated and dysfunctional (see Figure 1).

It is important to note that “integrated” and “functional” can have relative meanings. The model of the self-concept change concerns the population of people with different disturbances. In this study I investigated patients with diagnosis of neurosis or personality disorder. This is why “integrated” and “functional” should be understood as “closer” to the healthy end of the continuum among patients receiving psychotherapy.

#### Four phases of accommodation

In the process of self-concept change through accommodation one can distinguish four theoretical phases. The first is the initial state of equilibrium, the second is the period of increasing disintegration as a result of incoming elements and when new, competing self-image emerges. The third is the process of reintegration when the new state of equilibrium starts to win and leads to greater self-concept unity and the fourth phase is the dominance of a new self-concept schema when the self-concept is integrated. However, some people come to therapy with already existing very low level of self-concept unity; they are already disintegrated at the beginning of the therapy. I hypothesize that the process of accommodation among disintegrated patients starts with the third above-mentioned phase. The old self-schema is not unified, so it does not create a real competition for the new equilibrium and that is why it is possible to start directly with the process of reintegration. I predict that patients that are dysfunctional and disintegrated at the same time at the beginning of the treatment might experience only the second part of the “V” shape, so they might benefit from the monotonic increase of SCC.

#### Five shapes of SCC change

**Figure 2:**
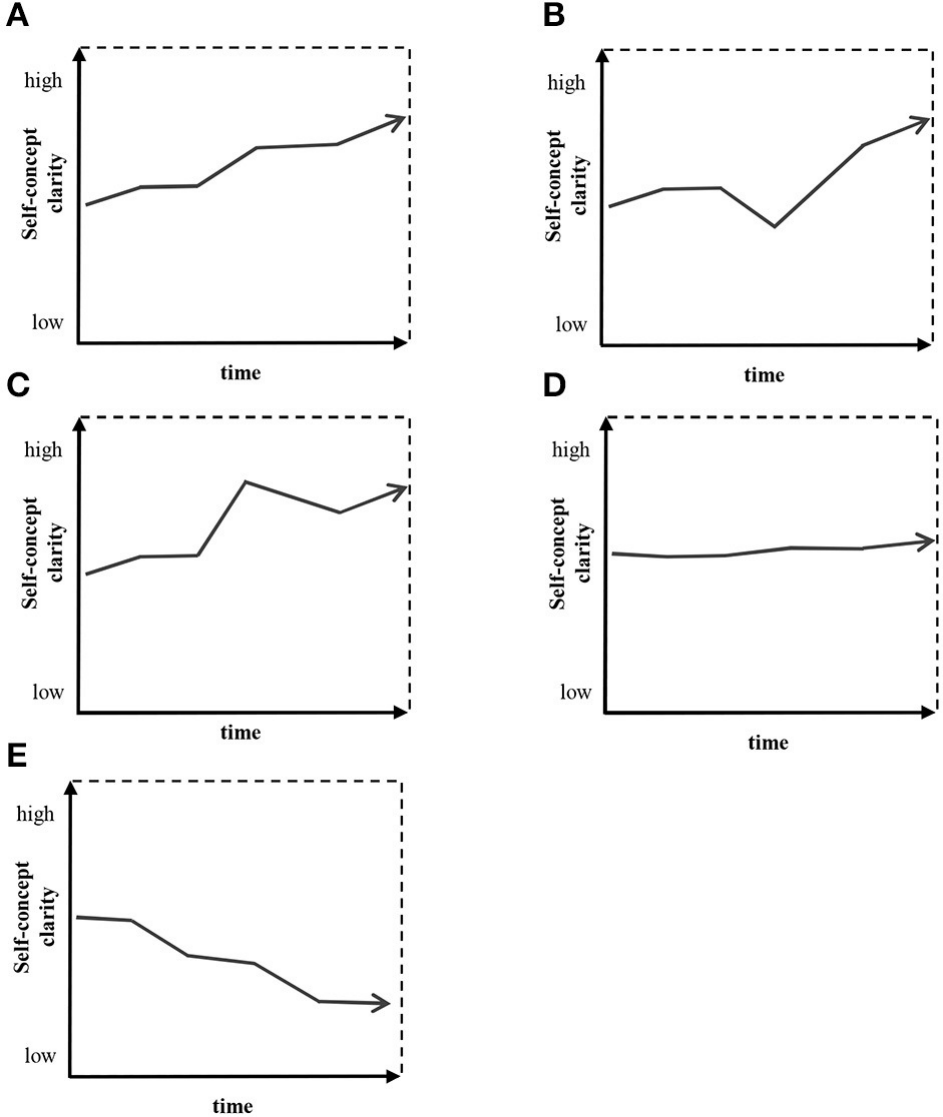
**Examples of self-concept-clarity shapes. (A)** Monotonic increase, **(B)** “V” shape, **(C)** discontinuous change except “V” shape, **(D)** plateau, **(E)** monotonic decrease.

In the model of self-concept change in psychotherapy, the focus is on the monotonic increase of SCC and “V” shape. It is worth noting that a “V” shape was already a matter of interest of psychotherapy researchers in the context of psychopathological symptoms (Hayes et al., 2007a,b) and therapeutic alliance (Stiles et al., 2004). But it is important not to forget that there are other probable shapes of change (Sobański, 2004). There are five shapes of SCC change that have been proposed: (1) monotonic increase, (2) “V” shape, (3) discontinuous change except “V” shape, (4) plateau, and (5) monotonic decrease (see Figure 2). These shapes of change were chosen as they encompass all the possible ways of change. They are a more detailed description of classical dichotomy between continuous change (monotonic increase, monotonic decrease) and discontinuous change (“V” shape and discontinuous change except “V” shape; Hayes et al., 2007b). It also takes into account the process of stable stagnation during therapy (plateau) (Sobański, 2004).

#### Hypotheses

**Figure 3:**
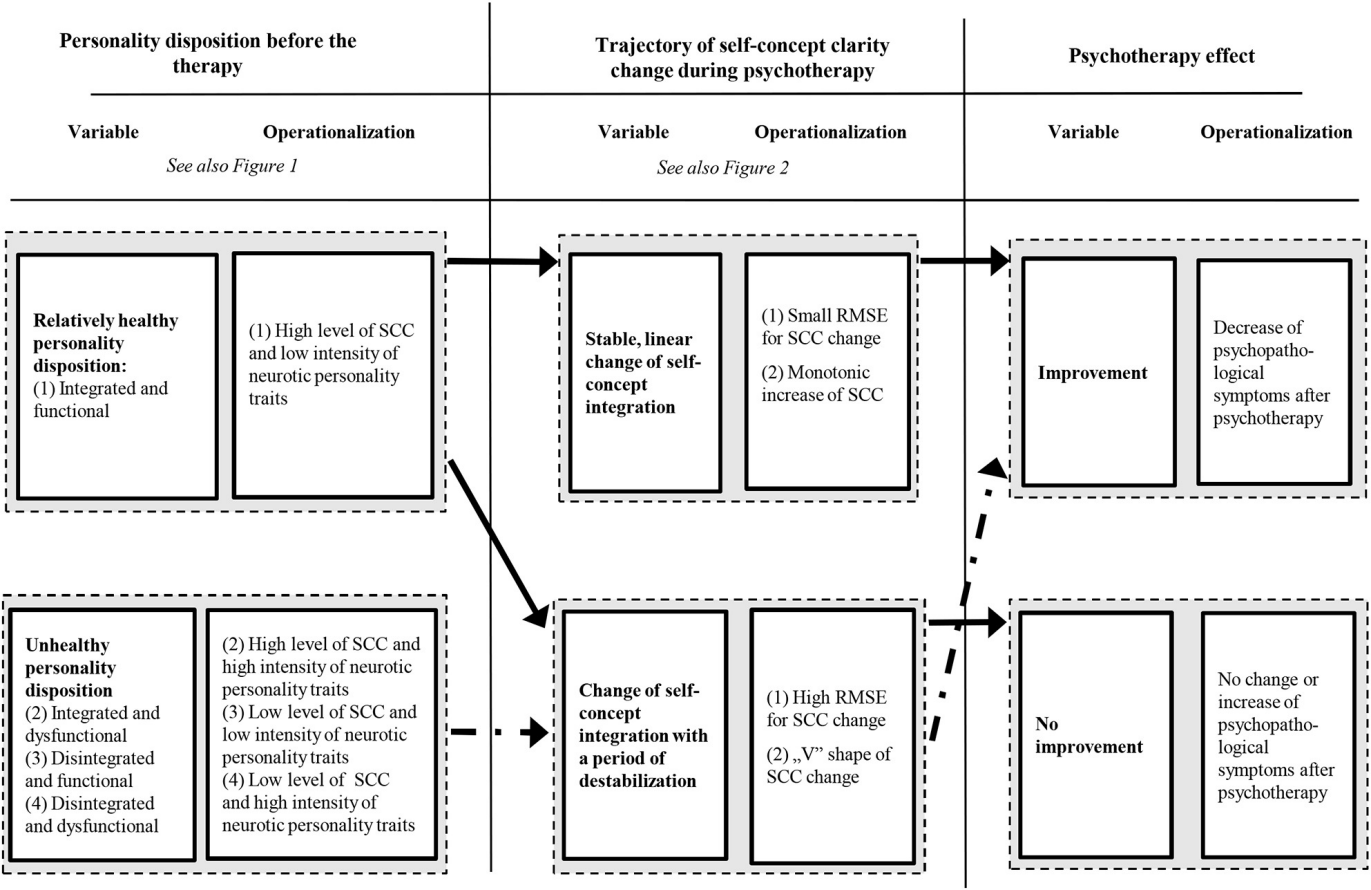
**Summary of the hypotheses: the relations between the personality dispositions, trajectory of the self-concept clarity change and the psychotherapy outcome**.

It is hypothesized that patients with an unhealthy structure of the self-concept will need a period of destabilization (SCC change in a form of a “V” shape or fluctuations). However, clients with relatively healthy personality dispositions benefit from a stable, linear change in the self-concept. Figure 3 depicts a summary of the hypotheses that are operationalized as follows:

1. There is an interaction between (1) the integrated and functional patients and (2) the remaining three groups of personality dispositions (integrated and dysfunctional, disintegrated and functional, and disintegrated and dysfunctional), concerning the relation between the SCC fluctuation (root mean square error of the SCC trajectory—SCC RMSE) and the magnitude of neurotic symptoms change from pre-therapy to post-therapy measurement. There is a negative correlation between SCC RMSE and the change of symptoms among the integrated and functional patients and positive correlation in the remaining three groups.
2. There is an interaction between (1) the integrated and functional patients and (2) the remaining three groups of personality dispositions (integrated and dysfunctional, disintegrated and functional, and disintegrated and dysfunctional), concerning the relation between monotonic increase and the “V” shape of SCC change, and the magnitude of neurotic symptoms change from pre-therapy to post-therapy measurement. The monotonic increase of SCC is more beneficial and the “V” shape is less favorable for the integrated and functional in comparison to the remaining three categories of personality dispositions.
  Moreover, it is specifically expected that:
  2a. Among the integrated and functional patients with a symptomatic improvement, there is a bigger frequency of monotonic increase of SCC than among the integrated and functional patients with no symptomatic improvement.
  2b. Among the integrated and dysfunctional patients with a symptomatic improvement, there is a bigger frequency of “V” shapes of SCC change than among the integrated and dysfunctional patients with no symptomatic improvement.
  2c. Among the disintegrated and dysfunctional patients with a symptomatic improvement, both the frequency of monotonic increase and “V” shape of SCC change is higher than among the disintegrated and dysfunctional patients with no symptomatic improvement.
  2d. Among all four groups of personality disposition with a symptomatic improvement, there is a smaller frequency of monotonic decrease of SCC than among these groups with no symptomatic improvement.

## Method

The research was approved by the ethics committee of the Faculty of Psychology, University of Warsaw, and all the participants gave a verbal informed consent. Participant consent was documented by the researchers in a special file. The informed consent was verbal because it used to be a standard procedure at the time when the study was conducted and it was approved by the ethics committee of the Faculty of Psychology, University of Warsaw. All the participants had a full capacity to consent, confirmed by the psychiatrist or psychologist, both employees of the hospitals where the study was conducted. No surrogate consent procedure was needed neither was one implemented.

### Participants

This research is based on a correlation model and was initially carried out on a group of 105 patients from three therapeutic units: 66 patients in the day-treatment setting and 39 in residential ward of psychiatric hospitals. Finally, full data were available for 85 patients of whom 27 (32%) were in-patients. All the analyses were conducted on the data received from this sample (*N* = 85). Twenty patients terminated their psychotherapy prematurely. This rate of dropout (19%) can be considered as common (Wierzbicki and Pekarik, 1993). Among the patients who terminated prematurely, 16 were women and four were men. All of the participants were Caucasian. The majority of the participants were women (*n* = 67, 79%). The mean age was 35 (*SD* = 10). Sixty two percent (*n* = 52) of the patients were students or people with higher education (see also Table 1).

**Table 1:** **Ns, proportions, means, SDs, and tests of differences between different personality dispositions for demographic and personality variables at Time 1 and therapy duration and medication**.

**Variable**		**Whole sample (*N* = 85)**		**Integrated and functional (*N* = 22)**		**Integrated and dysfunctional (*n* = 21)**		**Disintegrated and functional (*n* = 10)**		**Disintegrated and dysfunctional (*n* = 32)**				
		***N***	**%**	***N***	**%**	***N***	**%**	***N***	**%**	***N***	**%**	**χ*^2^***	***p***	***df***
Sex	Women	67	79%	17	77%	17	81%	9	90%	24	75%	1.18	0.773	3
	Men	18	21%	5	23%	4	19%	1	10%	8	25%			
Education	Primary	1	1%							1	3%	8.87	0.449	9
	Secondary	30	35%	9	41%	11	52%	1	10%	9	28%			
	Students	15	18%	2	9%	3	14%	3	30%	7	22%			
	Higher	37	44%	10	45%	7	33%	5	50%	15	47%			
	*Missing data*	*2*	*2%*	*1*	*5%*			*1*	*10%*					
Diagnosis	Neurosis	49	58%	14	64%	16	76%	4	40%	15	47%	15.26	0.084	9
	Personality disorder	16	19%	1	5%	1	5%	4	40%	10	31%			
	Neurosis and personality disorder	18	21%	7	32%	3	14%	2	20%	6	19%			
	Eating disorders	2	2%	0	0%	1	5%	0	0%	1	3%			
Medication	Medicated	52	61%	12	67%	12	56%	5	56%	23	56%	2.05	0.563	3
	Not medicated	18	21%	4	24%	4	17%	4	17%	6	17%			
	*Missing data*	*15*	*18%*	*6*	*9%*	*5*	*27%*	*1*	*27%*	*3*	*27%*			
		***M***	***SD***	***M***	***SD***	***M***	***SD***	***M***	***SD***	***M***	***SD***	***F***	***p***	**η**^2^
Age		35	10	39	11	36	10	31	9	32	8	3.01	0.035	0.10
Therapy duration in weeks		9	2	9	2	9	2	8	2	9	2	0.04	0.753	0.01
Self-concept clarity (SCCS)		35	10	45	8	41	5	28	3	26	5	58.50	*p* < 0.001	0.68
Symptoms of neurosis (KS-II)		296	99	233	85	315	99	260	104	339	84	6.72	*p* < 0.001	0.20
Neurotic personality traits (KON-2006)		42	24	17	11	54	15	18	10	60	15	56.88	*p* < 0.001	0.68

The nosological diagnosis was made by certified psychiatrists and psychologists based on the ICD-10 criteria. Most of the patients had diagnoses of neurosis (F40–48), personality disorders (F60–69), or both; two patients had diagnoses of eating disorders (F50). Among the neurotic disorders the most frequent diagnoses were “Other anxiety disorders” (F41, *n* = 39), “Reaction to severe stress, and adjustment disorders” (F43, *n* = 10), “Phobic anxiety disorders” (F40, *n* = 7), and “Obsessive-compulsive disorder” (F42, *n* = 7). Twenty-three patients were diagnosed with specific personality disorder (F60), 10 patients with mixed personality disorders (F61), and one patient with “Habit and impulse disorder” (F63).

### Therapists

The therapy was offered in the three different facilities chosen for the investigation and was conducted by 13 psychotherapists, 10 women, and three men. Eleven were psychologists and two were psychiatrists. Eight had finished their psychotherapy training, while six were still training; six were educated in psychodynamic approach, two in CBT, two in systemic psychotherapy, two in eclectic psychotherapy, and one in psychodrama. The amount of professional experience varied from 2 to 32 years with an average of 10.7 (*SD* = 9.6).

### Therapeutic program

The therapy was conducted in three institutions financed by state health insurance. The broad spectrum of places where the psychotherapy is offered was chosen to guarantee high external validity of the study. All patients participated in intensive time-limited group psychotherapy (routinely conducted by two therapists, 7.5 h of psychotherapy per week, approximately 12 patients per group) and other therapeutic activities (14 h per week of psychoeducational workshops, art therapy, psychodrama, gymnastics, and relaxation) of 6–12 weeks' duration. All three programs were eclectic and many activities were similar, but still there were some important differences. Altogether the data was collected from seven psychotherapeutic groups. In one of the institutions, the group psychotherapy was conducted using a psychodynamic approach (three open groups, *N* = 27), the second used CBT (two open group, *N* = 32), and the third engaged in an eclectic therapeutic mix greatly influenced by psychodrama (two closed groups, *N* = 26).

### Measures

#### Self-concept clarity

SCC was measured by the Polish adaptation of the Self-Concept Clarity Scale (SCCS; Campbell et al., 1996), made by Suszek (2002). The scale consists of 12 statements concerning one's perception of the self-concept, e.g., “My beliefs about myself seem to change very frequently” or “In general, I have a clear sense of who I am and what I am.” The statements are assessed on a 5-point Likert-type scale with anchors from 1 “strongly disagree” to 5 “strongly agree.” The Polish version of the questionnaire is characterized by satisfactory internal consistency reliability (Cronbach's alpha = 0.88, *N* = 230; Styła, 2011). Test-retest reliability with a 3-month interval is also satisfactory [*r*_(23)_ = 0.92, *p* < 0.001; Suszek, 2002]. The Polish version of the measure is also characterized by a satisfactory concurrent and convergent validity. It correlates negatively with measures of self-concept pluralism, self-concept incoherence, psychopathological symptoms and positively with self-esteem (Styła, 2011).

#### Intensity of neurotic symptoms

The Symptoms' Questionnaire KS-II (Aleksandrowicz and Sobański, 2001) was administered, which comprises 85 items. Participants were asked to rate the intensity of a given neurotic symptom within the last 2 weeks on a four-point scale (0, a, b, c) with anchors 0 “if the given symptom did not exist in the last 2 weeks” and c “if the given symptom was highly disturbing in the last 2 weeks.” The measure was standardized on the results of 742 people; 593 were people diagnosed with neurosis, personality disorders, or both and 149 were healthy controls. The internal consistency reliability of the questionnaire is satisfactory (Cronbach's alpha = 0.97, *N* = 165; Styła, 2011). The criterion and convergent validity of the KS-II is also satisfactory. It effectively differentiates population of patients with neurotic disorders from a healthy control group (Aleksandrowicz and Sobański, 2001). There is also a positive relationship between the results in KS-II and the Neurotic Personality Questionnaire KON**-**2006 (Styła, 2011).

#### Intensity of neurotic personality traits

Intensity of personality traits connected with occurrence and persistence of neurotic disorders was measured by the Neurotic Personality Questionnaire KON**-**2006 (Aleksandrowicz et al., 2009). In this questionnaire, the subjects are asked to answer 235 questions on a two-level scale (yes/no), e.g., “It is easy to hurt me” or “One should avoid strong emotions, they are too exhausting.” There are 24 subscales of the questionnaire. The measure was standardized on the results of 1314 people: 794 patients (before treatment) diagnosed with neurosis, personality disorders, dysthymia, and eating disorders and 520 healthy controls. The reliability of the questionnaire is satisfactory (Cronbach's alpha = 0.92). Test-retest reliability was good with a few hours' interval (r_(74)_ = 0.89, *N* = 76]. KON-2006 is also characterized by a satisfactory criterion and convergent validity. There is a significant difference in KON-2006 scores between patients diagnosed with neurotic disorders and persons from a healthy control group (Aleksandrowicz et al., 2009). There is also a positive correlation between results in KON-2006 and neurotic symptoms measured by The Symptoms' Questionnaire KS-II (Styła, 2011).

### Procedure

Participants filled in SCCS, Symptoms' Questionnaire KS-II, and Neurotic Personality Questionnaire KON-2006 at the beginning and at the end of the course of psychotherapy. SCCS and KS-II was also administered every 2 weeks during psychotherapy. The total number of measurements varied from 4 to 9 (*M* = 5.8, *SD* = 1.1). At all measurements the participants also filled in a scale SN-Ja-7 (Self-Incoherence Scale; Styła et al., 2010) that assesses Self-Concept Differentiation (Donahue et al., 1993). However, the results for this measure were not included in the current paper, as it would go beyond the scope of this article.

### Operationalization of the variables

In the current study the following variables need special attention: personality disposition, SCC fluctuation, shape of SCC change, and the indicators of psychotherapy effectiveness.

The group of 85 patients was divided into four personality disposition categories based on the following procedure. At the beginning, the method described by Jacobson and Truax (1991) was used to calculate the threshold for the group with integrated self-structure using the distributions of SCC in the healthy and dysfunctional populations and the data available from the literature. In the study of Roepke et al. (2011), the mean level of SCC among the women with BPD was 23.76 (*SD* = 7.44) and in a healthy sample (students and employees of various professions) researched by Stucke (2002) the average SCC reached 44.88 (*SD* = 11.28). The midpoint of the SCC distributions for these two populations was 34 points. The individuals from the study that had 34 or more points on the SCCS in the first measurement were defined as “integrated” and those with 33 or less points in the same questionnaire were put in the group labeled “disintegrated.” In the second step, the median of the KON-2006 results measured at Time 1 in the “integrated” group (*n* = 43) was calculated. This computed value (media *n* = 34 points) was used to create the final four subgroups: (1) integrated and functional (*n* = 22), (2) integrated and dysfunctional (*n* = 21), (3) disintegrated and functional (*n* = 10), and (4) disintegrated and dysfunctional (*n* = 32). In the current study, “functional” personality disposition is operationalized as having less than 34 points in the KON-2006 questionnaire.

Indicator of the SCC fluctuations is a root mean square error (RMSE) that is a standard deviation of ratings around the logarithmic trajectory of SCC over the course of psychotherapy. The higher the RMSE for SCC change, the bigger the SCC fluctuations. This method of computing fluctuations based on RMSE has already been used in psychotherapy research (Stiles et al., 1990; Barkham et al., 1993; Stulz et al., 2007). In the current study, the logarithmic—not linear—trajectory was used because it proved to be better adjusted to the trajectory of SCC change; see details—(Styła, 2011). The formula for this indicator is as follows:

$$RMSE=\frac{\sum_{i=1}^{k}(Y_{i}-Y_{i}^{\prime }{)}^{2}}{k}$$

*Y*_*i*_, score of the subject in SCCS in *i* measurement; *Y*${}_{i}^{\prime }$, expected score of the subject in SCCS in *i* measurement based on logarithmic trajectory; *k*, the number of measurements with the SCCS.

There were five shapes of SCC change that were proposed to single out: (1) monotonic growth, (2) “V” shape, (3) discontinuous change except “V” shape, (4) plateau, and (5) monotonic decline. The shapes of change are defined based on the reliable change index (RCI; Jacobson and Truax, 1991). The RCI value computed for a 95% level of confidence for the SCCS is 3.48. “Monotonic growth” is defined as occurrence of at least one increase in a score greater than 3.48 points from one measurement to the second following measurement in SCCS. No reliable decrease of SCC between two measurements can be observed. “V” shape is defined as occurrence of one decrease in a score bigger than 3.48 points from one measurement to the second following measurement in SCCS and then followed immediately with a reliable increase of SCC. “Discontinuous change except “V” shape” is defined as all the trajectories when at least one reliable increase and one reliable decrease of the SCC are observable but it is not the case for “V” shape. “Plateau” is defined as a situation when no reliable change of SCC from one measurement to the second has occurred. “Monotonic decline” is defined as occurrence of at least one reliable decrease from one measurement to the second following measurement in SCCS, while no reliable increase is observable.

The effectiveness was operationalized in a twofold manner. “Change of symptoms” was calculated by subtracting the result of the last measurement (post-therapy) of the Symptoms' Questionnaire KS-II from the first (pre-therapy). Positive values mean that the magnitude of symptoms of neurosis was smaller after therapy. All change-of-symptoms values were also categorized into two groups: “improvement” and “no improvement.” Improvement was defined as a change of symptoms greater than RCI value (above 56.6 points). The “no improvement” group included all other results.

### Statistical analyses

The results are based on clustered data. As participants were patients from three different therapeutic units and seven therapeutic groups that were conducted in these units, one can expect that membership in one unit or another and to one group or another is an important feature of the study. To ensure the accuracy of the analysis, multilevel modeling was applied with three levels of analysis (Heck et al., 2010). One of the analysis that was taken into consideration was also growth mixture modeling (GMM) that Stulz et al. (2007), for example, has used in research of shapes of change in psychotherapy. However, the sample size of this study is too limited to apply this method with success (Muñoz and Acuña, 1999; Maxwell et al., 2008). Moreover, the confirmatory analysis approach used in this article seems to be better adjusted than an exploratory statistical analyses (like GMM) to test the hypotheses of the proposed theoretical model of the self-concept change in psychotherapy.

## Results

As reported elsewhere (Styła, 2011), analyses of pre-post scores of the Symptoms' Questionnaire KS-II suggested significant decrease of neurotic symptoms after therapy.

### Four personality dispositions

To empirically validate the proposed four groups of personality dispositions, *k*-mean clustering was performed on the results of SCCS and Neurotic Personality Scale KON-2006 at Time 1. The computed four clusters were compared with the four categories received using the median split described above. The Cramer's V statistic confirmed that the two categorizations were probably measuring the same concept, Cramer's V(9, *N* = 85) = 0.75, *p* < 0.001. This is one of the reasons that substantiates creating the proposed categorization (based on the median split) and using it in further statistical analyses (De Coster et al., 2011).

Table 1 presents basic information on the whole sample and separately on the sub-groups of different personality dispositions that were created based on criteria presented above. The differences in sex, educational level, medication, and therapy duration between these four groups with different personality dispositions are not statistically significant. People in the (1) integrated and functional and (2) integrated and dysfunctional groups are on average older than in the (1) disintegrated and functional and (2) disintegrated and dysfunctional groups.

Differences shown in Table 1 concerning the SCC and neurotic personality traits are due to the criteria of classification of these personality disposition subgroups. Moreover, in the (1) integrated and functional and (2) integrated and dysfunctional groups there is a greater frequency of people with diagnosis of neurosis, while in the (1) disintegrated and functional and (2) disintegrated and dysfunctional groups more patients are diagnosed with a personality disorder. However, the differences concerning the diagnoses between the (1) integrated and functional and (2) integrated and dysfunctional as well as between (1) disintegrated and functional and (2) disintegrated and dysfunctional are not statistically significant. Convergent evidence for the presented classification can be seen in the analysis of differences between the groups as it concerns the initial level of psychopathological symptoms. The results of ANOVA with four personality dispositions as a between-subject factor indicate that there are statistically significant differences between the groups in the initial level of neurotic symptoms, *F*_(3, 81)_ = 6.72, *p* < 0.001, η^2^ = 0.19. *Post-hoc* analysis (Tukey's HSD test) suggests that there is a statistically significant difference in the initial level of neurotic symptoms between the (1) integrated and functional (*M* = 233, *SD* = 85) and (2) integrated and dysfunctional (*M* = 315, *SD* = 99) groups (*p* < 0.05), and between the (1) integrated and functional (*M* = 233, *SD* = 85) and (2) disintegrated and dysfunctional (*M* = 339, *SD* = 84) groups (*p* < 0.001).

### Fluctuations of the self-concept clarity and the effectiveness of psychotherapy (hypothesis 1)

To ensure the accuracy of the analysis, multilevel modeling was applied with three levels of analysis—three therapeutic units (level 3), seven therapeutic groups (level 2) and individual differences (level 1). The “personality disposition” variable in this analysis consists of two groups: (1) integrated and functional (*n* = 22) and (2) the remaining three personality dispositions (integrated and dysfunctional, disintegrated and functional, and disintegrated, and dysfunctional) (*n* = 63). The change of neurotic symptoms after psychotherapy was computed as follows: for every participant the measured intensity of neurotic symptoms after psychotherapy was subtracted from the intensity of neurotic symptoms at the beginning of the treatment.

In the first step of the analysis, the null model (the model without predictors) was examined. Four parameters were estimated: the grand mean of change of neurotic symptoms in the sample (the intercept), the variance of means of neurotic symptoms in seven therapeutic groups (the variance of intercepts), the variance of means of neurotic symptoms in three units (the variance of intercepts) and the residual. Table 2 presents estimates of covariance parameters.

**Table 2:** **The variance components of the change in the intensity of neurotic symptoms**.

**Parameter**	**Estimate**	**Wald**	***p***
Residual	7269.93	6.30^***^	0.001
Intercept group	581.72	0.59	0.553
Intercept therapeutic unit	106.91	0.14	0.892

Wald: test of significance of parameters; p: statistical significance;

*** *p < 0.001*.

The intercepts (means of the change in the intensity of neurotic symptoms) did not vary across the seven psychotherapeutic groups and the three units. This result suggests that it would be pointless to continue further analyses with the level 2 and level 3 (Heck et al., 2010). Only residual, which is an estimate of individual differences in the change of the intensity of neurotic symptoms, was statistically significant. As a consequence, in the subsequent analysis, only level 1 (individual differences) was taken into account. Level 2 (therapeutic groups) and level 3 (units) was not included.

In the next model, the four parameters of fixed effects were estimated: the intercept (the grand mean), the SCC fluctuation, personality disposition and the interaction between the SCC fluctuation and personality disposition. The whole model explains 12.9% of the variance. As seen in Table 3, the SCC fluctuation is a statistically significant predictor of the outcome. It explains 3.1 % of the variance. In accordance with hypothesis 1, the interaction between the personality disposition and the SCC fluctuations also significantly predicted the outcome. It explained 7.9% of the variability. The value of the interaction term (−48.49) means that the slope of the regression line with the SCC RMSE as a predictor and change of neurotic symptoms as a dependent variable is more gentle among the integrated and functional patients than in the three remaining groups. The analysis of the nature of the interaction suggests that the functional and integrated patients do not benefit from SCC fluctuations, *r*_(20)_ = −0.22, *p* > 0.05, while among the three remaining groups of patients there is a significant correlation between the two variables, *r*_(61)_ = 0.42, *p* < 0.001.

**Table 3:** **Estimate of fixed effects**.

**Parameter**	**Estimate**	***T***	***p***	**Explained variance**
Intercept	1.92	0.01	0.922	–
SCC RMSE	29.76	3.31^**^	0.001	3.1%
PD-FI^a^	61.25	1.69	0.095	1.9%
PD-FI ^*^ SCC RMSE^b^	−48.49	−2.73^**^	0.008	7.9%

a *PD-FI = two groups of personality dispositions—(1) integrated and functional vs. (2) integrated and dysfunctional, disintegrated and functional, and disintegrated and dysfunctional*.

b *SCC RMSE = fluctuations of the self-concept clarity (RMSE = Root Mean Square Error)*.

*Wald, test of significance of parameters; p, statistical significance*.

** *p < 0.01*.

### Shape of the self-concept clarity change and the effectiveness of psychotherapy (hypothesis 2)

**Figure 4:**
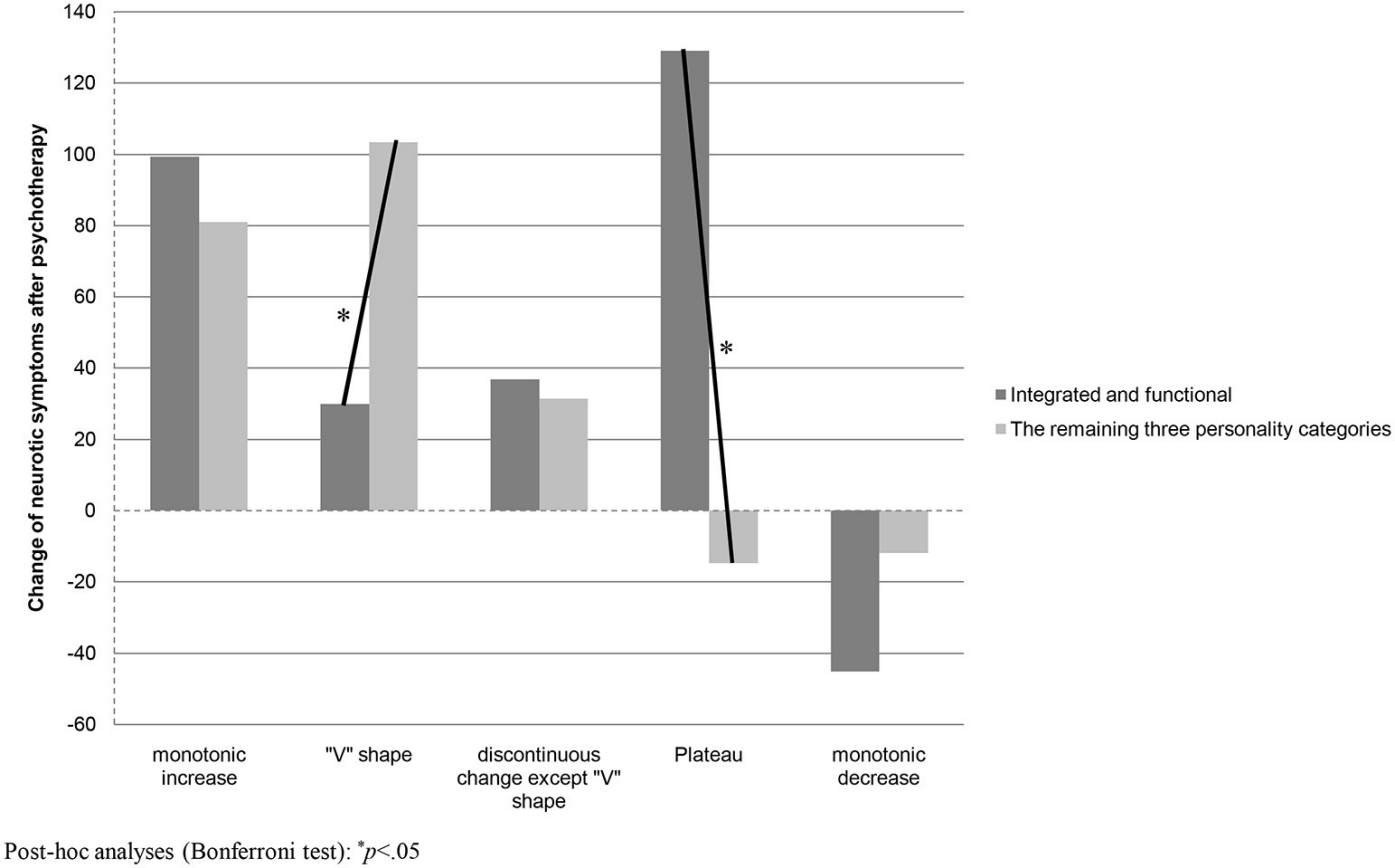
**Change of neurotic symptoms after psychotherapy measured by the Symptoms' Questionnaire KS-II (positive values mean improvement) among two groups of personality disposition and in five shapes of SCC change**.

ANOVA with personality disposition (functional and integrated/the remaining three categories of personality disposition) and the type of shape of SCC change during psychotherapy (monotonic increase, “V” shape, discontinuous change except “V” shape, plateau, and monotonic decrease) were used as two between-subject factors to examine the interactional effect between (1) the personality disposition and (2) shape of SCC change on the neurotic symptom change after psychotherapy. As seen in Table 4, the main effect of the shape of SCC change was statistically significant, *F*_(1, 75)_ = 4.09, *p* < 0.01, η^2^ = 0.18, while the personality disposition x shape of SCC change interaction was significant on the level of statistical tendency, *F*_(4, 75)_ = 2.25, *p* < 0.07, η^2^ = 0.11. The main effect of personality disposition was not statistically significant, *F*_(1, 75)_ = 0.03, *p* > 0.05, η^2^ = 0.00. A post-hoc analysis (Bonferroni test) was used to analyse the nature of the interactional effect (see also Figure 4). It was found that the improvement in neurotic symptoms was statistically higher among the integrated and dysfunctional, disintegrated and functional, and disintegrated and dysfunctional experiencing a “V” shape of SCC change (*M* = 103, *SD* = 87) than among the integrated and functional (*M* = 30, *SD* = 116) (*p* < 0.05). The difference between the two analyzed groups concerning the monotonic SCC increase was not statistically significant, but it appeared that the improvement in neurotic symptoms was higher among the functional and integrated patients experiencing a plateau shape of SCC change (*M* = 129, *SD* = 58) than among the remaining three personality disposition categories (*M* = −15, *SD* = 68).

**Table 4:** **Summary of Two-factor variance analysis (shapes of self-concept clarity change and personality dispositions) with change of neurotic symptoms as a dependent variable**.

**Factors**	***df***	***F***	**η*^2^***	***P***
Shape of SCC change	4	4.09	0.18	0.005
PD-FI^a^	1	0.03	0.00	0.583
Shape of SCC change ^*^ PD-FI	4	2.25	0.11	0.071

a *PD PD-FI = two groups of personality dispositions—(1) integrated and functional vs. (2) integrated and dysfunctional, disintegrated and functional, and disintegrated and dysfunctional*.

### Shape of the self-concept clarity change and the effectiveness of psychotherapy (hypotheses 2a, 2b, 2c, and 2d)

A likelihood ratio was used to analyse the frequencies of different shapes of the SCC change in the group of patients who experienced significant symptomatic improvement compared with those who did not improve (significant deterioration or no significant change). (When the expected frequencies are low, likelihood ratio is recommended as a better alternative of χ^2^ with Yates continuity correction (Field, 2013). Likelihood ratio (Lχ^2^) is also based on the χ^2^ distribution). The summary of all analyses is presented in Table 5.

**Table 5:** **Summary of the frequencies: shapes of self-concept clarity change and psychotherapy effectiveness categorizations**.

**Group**	**Shape of self-concept clarity change**	**Improvement**	**No Improvement**	**Lχ^2^**	***p***
Whole sample (*n* = 85)	Monotonic increase	10/39 (26%)	5/46 (11%)	3.19	0.074
	“V” shape	20/39 (51%)	9/46 (20%)	9.59	0.002
	Discontinuous change except “V” shape	7/39 (18%)	13/46 (28%)	1.27	0.26
	Plateau	2/39 (5%)	4/46 (9%)	0.41	0.52
	Monotonic decrease	0/39 (0%)	15/46 (33%)	21.13	< 0.000
Integrated and functional (*n* = 22)	Monotonic increase	3/11 (27%)	0/11 (0%)	4.63	0.03
	“V” shape	4/11 (36%)	3/11 (27%)	0.21	0.65
	Discontinuous change except “V” shape	2/11 (18%)	3/11 (27%)	0.26	0.61
	Plateau	2/11 (18%)	0/11 (0%)	2.97	0.08
	Monotonic decrease	0/11 (0%)	5/11 (45%)	8.42	0.004
Integrated and dysfunctional (*n* = 21)	Monotonic increase	1/8 (13%)	3/13 (23%)	0.38	0.54
	“V” shape	6/8 (75%)	3/13 (23%)	5.64	0.02
	Discontinuous change except “V” shape	1/8 (13%)	2/13 (15%)	0.03	0.85
	Plateau	0/8 (0%)	2/13 (15%)	2.05	0.15
	Monotonic decrease	0/8 (0%)	3/13 (23%)	3.18	0.07
Disintegrated and functional (*n* = 10)	Monotonic increase	0/3 (0%)	1/7 (14%)	0.76	0.38
	“V” shape	1/3 (33%)	2/7 (29%)	0.02	0.88
	Discontinuous change except “V” shape	2/3 (67%)	1/7 (14%)	2.66	0.10
	Plateau	0/3 (0%)	1/7 (14%)	0.76	0.38
	Monotonic decrease	0/3 (0%)	2/7 (29%)	1.63	0.20
Disintegrated and dysfunctional (*n* = 32)	Monotonic increase	6/17 (35%)	1/15 (7%)	4.20	0.04
	“V” shape	9/17 (53%)	1/15 (7%)	8.89	0.003
	Discontinuous change except “V” shape	2/17 (12%)	7/15 (47%)	4.98	0.03
	Plateau	0/17 (0%)	1/15 (7%)	1.55	0.21
	Monotonic decrease	0/17 (0%)	5/15 (33%)	8.64	0.003

These analyses indicate that the “V” shape is statistically more frequent among the group “improvement” than in the group “no improvement,” Lχ^2^(1, *N* = 85) = 9.59, *p* < 0.01. The contrary effect is seen for monotonic decrease. Zero out of 39 patients who improved had a monotonic decrease of SCC, while 15 out of 46 in the “no improvement” group (33% of the total) had this shape of SCC change. This difference has a high level of statistical significance, Lχ^2^(1, *N* = 85) = 21.13, *p* < 0.001.

The monotonic increase was more frequently in the “improvement” group than in the “no improvement” group among patients described as integrated and functional, Lχ^2^(1, *N* = 22) = 4.63, *p* < 0.05, and among disintegrated and dysfunctional, Lχ^2^(1, *N* = 32) = 4.20, *p* < 0.05.

The “V” shape was more frequently in the “improvement” group than in the “no improvement” group among patients described as integrated and dysfunctional, Lχ^2^(1, *N* = 21) = 5.64, *p* < 0.05, and among disintegrated and dysfunctional, Lχ^2^(1, *N* = 32) = 8.89, *p* < 0.01.

## Discussion

In the current study I investigated individual trajectories of the SCC during psychotherapy among patients diagnosed with neurosis and personality disorders. The multilevel analysis (the null model) suggested that the three different therapeutic units (level 3) or the membership in one of the seven psychotherapeutic groups that were conducted on the three units (level 2) do not add any further information to the studied phenomena. The individual differences (level 1) proved to be the only level of analysis that should be taken into account.

The statistical analyses confirmed that SCC fluctuations are correlated with the drop in neurotic symptoms at the end of the treatment. However, the strength of the relation was small: 3.1% of the variation of the symptom outcome can be explained by SCC fluctuation. As postulated by the model of self-concept change SCC fluctuations proved to be beneficial for the integrated and dysfunctional, disintegrated and functional, and disintegrated and dysfunctional, but there was no relation to the psychotherapy outcome among the integrated and functional patients (partially confirming hypothesis 1). This interactional effect accounted for 7.9% of the variability. The magnitude of the effect seems to be small, but when compared to the results of other studies on other interactional models in psychotherapy (e.g., Aptitude × Treatment Interaction (ATI) model) it proves to be a significant effect (Beutler et al., 2005). Moreover, the strength of the direct relation between SCC fluctuations and symptom outcome among the integrated and dysfunctional, disintegrated and functional, and disintegrated and dysfunctional patients is comparable (*r* = 0.42) to the effects obtained in other studies on self-concept variability (Cummings et al., 2012).

A similar pattern of results was obtained when the shapes of SCC change were analyzed. The “V” shape of SCC change was observed among 34% of the sample. It was proven to be more frequent among those patients who benefited from the psychotherapy (51%) than among the group that was not successful in symptom reduction (20%). Among the integrated and functional the “V” shape of SCC change was less effective therapeutically than among the remaining three personality dispositions, while the plateau shape of SCC trajectory was more effective among the integrated and functional group than among patients in the remaining three personality categories. This interactional effect explained 11% of outcome variance. As the effect was not statistically significant for the monotonic increase of SCC this result only partly confirms hypothesis 2. However, at the same time, hypothesis 2a was confirmed—monotonic SCC increase, but not “V” shape, was more frequent among integrated and functional patients who experienced symptom improvement than among the equivalent group who had no success. Moreover, the “V” shape of SCC change was characteristic for 75% of the integrated and dysfunctional patients who improved in comparison with 23% who experienced no improvement. This difference was statistically significant and confirmed hypothesis 2b. As hypothesized (hypothesis 2c), for the disintegrated and dysfunctional patients both monotonic SCC increase and “V” shape were related to symptom improvement. The monotonic SCC decrease was confirmed to be a strong predictor of psychotherapy failure. No single patient from the “improvement” group experienced this shape of SCC change, while one third of the patients with no improvement did (confirmation of hypothesis 2d).

These results give support for almost all of the hypotheses drawn from the model of self-concept change (for summary see Table 6). The only hypothesis that was not fully confirmed concerned the role of monotonic increase of SCC. It was not proven that this shape of change is more beneficial for the integrated and functional group than for the other categories of patients. A reason for that might be connected with the assumption that the monotonic shape of change does not reflect a process of assimilation in case of disintegrated patients. So, more important might be the result that monotonic increase of SCC was connected with psychotherapy success among the functional and integrated while the “V” shape was not—which constitutes evidence for a differential role of these two shapes of change for the functional and integrated patients. An unexpected result concerning the role of plateau shape of SCC trajectory might be also incorporated into the self-concept change model. In light of this result, one can argue that plateau may also be a sign of the assimilation process—small alterations in the self-schema might not necessarily increase the SCC change. However, one has to be cautious with this conclusion as it is difficult to differentiate, without any additional measures, the assimilation process from a situation when no changes occurred in the self-concept schema.

**Table 6:** **Summary of the study: hypotheses, results, and conclusions**.

**Hypothesis No**.	**The content of the hypotheses**	**Results**	**Conclusions**
#1	There is an interaction between (1) the integrated and functional patients and (2) the remaining three groups of personality dispositions^a^ concerning the relation between the SCC^b^ fluctuation (SCC RMSE^c^) and the magnitude of neurotic symptoms change from pre-therapy to post-therapy measurement	t –ratio with test of significance of parameters *p* < 0.01, explained variance = 7.9%	The hypothesis is partially confirmed. The statistically significant correlation between the SCC fluctuation and the psychotherapy outcome was observed only among the group of patients with unhealthy personality disposition
	There is a negative correlation between SCC RMSE and the change of symptoms among the integrated and functional patients	*r*_(20)_ = −0.22, *p* > 0.05	
	There is a positive correlation between SCC RMSE and the change of symptoms among the remaining three groups	*r*_(61)_ = 0.42, *p* < 0.001, explained variance = 17.6%	
#2	There is an interaction between (1) the integrated and functional patients and (2) the remaining three groups of personality dispositions concerning the relation between monotonic increase and the “V” shape of SCC change, and the magnitude of neurotic symptoms change from pre-therapy to post-therapy measurement	*F*_(4, 75)_ = 2.25, *p* < 0.07, explained variance = 11%	The hypothesis is partially confirmed. The difference was confirmed only for the “V” shape Moreover as the *p*-value amounts 0.07 this result needs further studies with greater sample size
	The monotonic increase of SCC is more beneficial for the integrated and functional in comparison to the remaining three categories of personality dispositions	Bonferroni test, *p* > 0.05	
	The “V” shape is less favorable for the integrated and functional in comparison to the remaining three categories of personality dispositions	Bonferroni test, *p* < 0.05	
#2a	Among the integrated and functional patients with a symptomatic improvement, there is a bigger frequency of monotonic increase of SCC than among the integrated and functional patients with no symptomatic improvement	Lχ^2^(1, *N* = 22) = 4.63, *p* < 0.05	The hypothesis is confirmed
#2b	Among the integrated and dysfunctional patients with a symptomatic improvement, there is a bigger frequency of “V” shapes of SCC change than among the integrated and dysfunctional patients with no symptomatic improvement	Lχ^2^(1, *N* = 21) = 5.64, *p* < 0.05	The hypothesis is confirmed
#2c	Among the disintegrated and dysfunctional patients with a symptomatic improvement, both the frequency of monotonic increase and “V” shape of SCC change is higher than among the disintegrated and dysfunctional patients with no symptomatic improvement	Lχ^2^(1, *N* = 32) = 4.20, *p* < 0.05 Lχ^2^(1, *N* = 32) = 8.89, *p* < 0.01	The hypothesis is confirmed
#2d	Among all four groups of personality disposition with a symptomatic improvement, there is a smaller frequency of monotonic decrease of SCC than among these groups with no symptomatic improvement	Lχ^2^(1, *N* = 85) = 21.13, *p* < 0.001	The hypothesis is confirmed

a *Remaining three groups of personality—integrated and dysfunctional, disintegrated and functional, and disintegrated and dysfunctional*.

b *SCC, Self-concept clarity*.

c *SCC RMSE, fluctuations of the self-concept clarity (RMSE = Root Mean Square Error)*.

The theoretical model and the findings are also partly in line with the results of Hayes and Strauss (1998) and Hayes et al. (2007a). Both the model of self-concept change and the conception of Hayes et al. (2007a) place emphasis on the notion of destabilization—a period of cognitive, affective, or behavioral turbulence. The important difference between these two concepts is that the model of self-concept change tries to address additionally the question “For what type of patients is the destabilization period optimal?” The results suggest that destabilization is optimal for the people with united self-concept and dysfunctional self-schemas and partly for the disintegrated and dysfunctional patients. The conclusion from this study that patients with different personality dispositions need different trajectories of change is supported by the results of the studies carried out by Polman et al. (2011) on a group of OCD patients and Vermote et al. (2011) who researched patients with severe personality disorders. The approach of matching different mechanism of change (and the techniques behind it) to the features of the patient can be also seen as an important way to enhance psychotherapy effectiveness (Beutler et al., 2005).

### Conclusions for the practice

The current study should be perceived as a preliminary report, but already some cautious conclusions for the practitioners might be formulated. First of all, the results suggest that a temporal destabilization of some patients concerning the self-concept should not be a cause of concern. Indeed, it can be interpreted as a sign of a good treatment prognosis. This relates especially to the patients who, at the beginning of the therapy, seemed to be sure about their view of themselves, but at the same time their beliefs about themselves were misleading. Secondly, the results suggest that the patients with unified self and quite healthy self-schemas might benefit from a process of psychotherapy without cognitive turbulence. Last, but not least, we can conclude that it is important to monitor the process of self-concept disintegration, so it does not last too long. If necessary, it seems justified to implement techniques that could lead to self-concept reintegration, or the therapy should be extended so the whole process of disintegration and reintegration can be completed.

### Limitations

There are limitations to this study. First of all, the criteria for the creation of the personality dispositions were—because of the limited size of the research group—based on the median split of the results of the KON-2006 questionnaire. Therefore, although the *k*-mean clustering and the analysis of the differences between the four personality dispositions (initial level of symptoms, and diagnosis between the integrated and disintegrated groups) suggest that this split is characterized by a convergent validity, one of the two values used in this study to create the four groups (34 points in the KON-2006 questionnaire) is bound to this specific sample. Future studies should address this problem, so that the features of the postulated groups of personality dispositions are precisely defined and clearly operationalized. It would help future research to be more comparable. Another topic that needs more research is the meaning of the proposed personality dispositions for clinicians. How can each of the distinguished groups, presented in the model of self-concept change, be understood in commonly used clinical terms?

The *p*-value for the interactional effect between (1) the personality disposition and (2) shape of SCC change on the neurotic symptom change after psychotherapy amounted 0.07. Further, analyses of this effect were conducted following the suggestion of many methodologists (e.g., Kline, 2004; Sullivan and Feinn, 2012) that when the effect size is significant it is recommended to continue analyses even when the *p*-value is greater than 0.05. However, this result should be treated cautiously and further studies on this issue based on bigger samples should be conducted. Based on the results of this study this effect would become significant when the sample had more than 102 participants.

Another limitation of the study is that it does not determine directly whether the processes of assimilation and accommodation are responsible for the observed monotonic growth and “V” shape of SCC change. Further studies should examine whether the small alteration in the self-schemas produces a gradual growth of SCC, while observable reconstruction of self-concept leads to the high-low-high pattern.

Moreover, interesting questions are left unanswered concerning the conclusions for practitioners. Are there techniques that lead to assimilation process that are especially appropriate for the integrated and functional patients? What techniques could lead to accommodation that should be optimal for the integrated and dysfunctional patients? We might imagine that the continuum of techniques in the psychodynamic psychotherapy from the supportive to the explorative end (Leichsenring et al., 2006) could form a framework for the future studies on the relation between techniques, shapes of SCC change, and personality dispositions.

Other limitations of the study arise because it is based on a naturalistic sample gathered under routine care conditions and that the patients participated in an intensive program based on many group forms of treatment (group psychotherapy with additional psychoeducational workshops, art therapy, psychodrama, gymnastics, and relaxation). Thus, it was not feasible to control the psychotherapeutic interventions, and detailed information about participants and therapists was lacking. The internal validity was limited, but the external validity was high. Moreover, the study was conducted among participants with diagnosis of neurosis and personality disorders, so the results cannot be extrapolated to different populations. Future research on different populations in more controlled settings is needed. Such replications and extension of the current study could reveal whether the predictions of the self-concept change model are also representative for other groups of patients. Furthermore the statistical analyses don't include the data gathered from the patients who prematurely dropped out what can be a source of biases. Finally, the nature of the study is correlational; that is why the conclusions about the impact of the changes in self-concept on the therapeutic outcome should be treated cautiously.

To the author's knowledge, this is the first attempt to explore the individual data on SCC trajectories through the psychotherapeutic process. Having in mind all the limitations listed above, the presented model opens a new and interesting area of research—examination of the trajectories of the self-concept change. Better understanding of the process of SCC change moderated by different personality dispositions might in future lead to the creation of integrative models that would allow psychotherapy to be designed and conducted in a more effective way.

### Conflict of interest statement

The author declares that the research was conducted in the absence of any commercial or financial relationships that could be construed as a potential conflict of interest.

## Footnotes

^1^A presentation of the dynamic systems theory would go beyond the scope of this article, which is why I focus only on the conclusions drawn from the work of van Geert (1998). All the readers interested in deepening the knowledge about this topic are encouraged to reach the classical book of Nowak and Vallacher (1998) or the presentation of dynamic systems theory in the context of psychotherapy done by Hayes and Strauss (1998).
